# Animal Welfare Centres: Are They Useful for the Improvement of Animal Welfare?

**DOI:** 10.3390/ani10050877

**Published:** 2020-05-18

**Authors:** Clive J. C. Phillips, Carla F. M. Molento

**Affiliations:** 1 Centre for Animal Welfare and Ethics, School of Veterinary Science, The University of Queensland, Gatton, QLD 4343, Australia; 2 Animal Welfare Laboratory, Animal Science Department, Federal University of Parana, Curitiba, R. dos Funcionarios 1540, Brazil; carlamolento@yahoo.com

**Keywords:** animal wellbeing, animal welfare, animal welfare science, centres of animal welfare

## Abstract

**Simple Summary:**

Animal welfare has only recently become the subject of scientific studies. Over the past 30 years, a body of scientists has emerged, initially in isolated groups in disparate universities but with some more recently aggregated into centres. Centres have developed a reputation for their research in specific fields, which may have allowed animal welfare knowledge to advance more rapidly than it would otherwise have done. We studied the scientific literature on animal welfare that has been published in journals, by both scientists in animal welfare centres and those outside of these centres. We found that the literature from centres was more likely to acknowledge industry funding, which seems to bring opportunities for the respective scientists to conduct more research in their field but may also make it difficult for those scientists to advocate animal welfare improvements if they conflict with industry objectives. We advocate strict standards for the operations of animal welfare centres that govern the ethics of their research into animal welfare.

**Abstract:**

Animal welfare has emerged as a scientific discipline only in the past 30 years, but a significant body of scientists has developed worldwide in this time. Over the past quarter century, several aggregations of scientists, centres of animal welfare, have become established. This can bring the benefits of the recognition of expertise and an opportunity to support animal industries in improving welfare, but it also brings the risk of scientists being influenced by these industries and failing to identify animal welfare problems as such. We conducted a bibliometric search of the scientific literature with the purpose of comparing the characteristics of publications on animal welfare that were or were not from animal welfare centres in academic institutions. We found that the number of publications on animal welfare from centres of animal welfare increased, initially, in the early 2000s and again in the last decade. Significant funding was obtained from the livestock industries for these centres. In a second search, we identified that only about 8% of scientific publications on animal welfare came from animal welfare centres, and the rest were mainly supported by funding sources other than the animal industries. It is concluded that the emergence of significant animal welfare centres, often with significant funding from industry, allows clusters of scientists to develop that could advance animal welfare knowledge more effectively than disparate scientists in isolated institutions. However, industry funding risks these scientists being aligned with industry goals that may not include animal welfare improvement to the extent required by the public. Further research to identify any ethical conflicts for scientists in animal welfare centres would be warranted.

## 1. Introduction

Animal welfare is a new and rapidly growing science. It has emerged in the last 30 years, with an exponential growth in the number of publications, in response to growing concern about the welfare of animals. Broom [1] charts its development as starting with the book *Animal Machines* by Ruth Harrison [2], published in 1964, which prompted the establishment of a committee in 1965 by the UK government, led by FR Brambell. This committee was tasked with investigating the welfare of animals kept under intensive livestock husbandry systems. The ensuing Brambell report paved the way for the widespread adoption of five key freedoms for farm animals [3], which became a set of guiding principles used throughout the world, by the farming industry, advocacy groups and scientists. Just one year later, in 1966, the Society for Veterinary Ethology was formed, which subsequently became the International Society for Applied Ethology in 1991 [4]. This cemented the study of the welfare of, for the most part, managed—as opposed to wild—animals as a recognised scientific discipline, all stemming from the concern of the public, enunciated by Harrison in her book, about the increasingly intensive farm animal management systems being used in the UK at the time. 

The new science needed journals in which to publish the results of scientific experiments in animal welfare [5]. In 1977, Elsevier launched *Animal Regulation Studies* sponsored by the then World Federation for the Protection of Animals, a journal which lasted until 1981. Another short-lived success was when The Humane Society of the United States produced the *International Journal for the Study of Animal Problems* in 1980, which lasted until 1983. It was continued until 1986 as annual volumes under another title: *Advances in Animal Welfare Science*. These initiatives show efforts to address the gap in scientific publications regarding animal welfare, and their short life spans may be a testimony to the difficulties in advancing animal welfare science, which was then a restricted field with few followers. These journals were quite possibly ahead of their time. In 1992, a journal entitled *Animal Welfare* was created, as an initiative of the Universities Federation for Animal Welfare (UFAW), an independent registered charity established in 1926 as the University of London Animal Welfare Society and changed to UFAW in 1938; this journal has been offering a steady contribution to animal welfare science since then. Currently, just one other journal includes animal welfare in its title, the *Journal of Applied Animal Welfare Science*, with its first volume published in 1998.

There emerged a group of scientists addressing these issues, most based in universities. Some of the scientists were based in veterinary schools and others in agriculture or animal science schools or research institutes, although, in general, the science was not well accepted in veterinary fields, despite its obvious relevance. Later, due to either the aggregation of these scientists into groups or to their increased individual organisation and strength, institutional animal welfare research units were founded, termed as centres, laboratories, institutes, units or groups (from now on referred to as centres), which suggested some permanence of their contribution and a common intrainstitutional and interinstitutional approach to be adopted. When the centres include the term “animal welfare” in their titles, it seems reasonable to expect special attention to animal welfare issues, with an animal-centred approach. However, we recognise that this is not always the case. In addition, centres with no reference to animal welfare in their titles may also perform animal welfare related activities, such as some centres focused on animal behaviour.

Animal welfare centres are considered to be research and teaching groups, as appropriate to institutions of learning such as universities. The first initiative in this direction is believed to have been the Animal Welfare Institute of the USA, established in 1951, which had publications in magazines and other non-scientific literature and thus cannot be considered to fulfil our definition of a centre, with research and teaching objectives. In 1978 emerged the first article from a university animal welfare centre, the University of Budapest veterinary school’s Animal Welfare Station [6]. The first articles to come out of the Hannover Veterinary School’s Institut Tierhygiene, Tierhaltung und Tierschutz emerged in 1988 [7], which was later to become a very productive centre. A prominent animal welfare research group in the Department of Veterinary Medicine, University of Cambridge started in 1986 with the appointment of Donald M. Broom as the Colleen Macleod Professor of Animal Welfare. Research on welfare concepts and principles and welfare issues in farms, laboratories and wild animals commenced. In 1987, Broom and James Serpell set up the Companion Animal Research Group, whose work included some behavioural studies on companion animals in areas other than welfare. In 1997, the name was changed to Animal Welfare and Human-Animal Interactions Group to better reflect the research activities. By 2006, work was also being done on human welfare and there was concern that “Human-Animal Interactions” might give the impression that humans are not animals, contrary to the general view in the group. Hence the title was changed to the Centre for Animal Welfare and Anthrozoology (CAWA). Anthrozoology refers to all interactions and relationships between humans and other animal species, and the group had for many years included the editor of the Journal Anthrozöos, Anthony Podberscek. 

Another key centre, the Animal Welfare Centre, started at the University of Melbourne in 1997, later renamed as the Animal Welfare Science Centre in 2003. Additionally in 1997, the Animal Welfare Program was established at the University of British Columbia, Canada, “program” being a term commonly used in North America for a centre of teaching and research activity. Then, in 1998, the Animal Welfare Science and Bioethics Centre was founded in Massey University. In Brazil, the first university centre to include the term animal welfare in its name was the Animal Welfare Laboratory, from the Federal University of Parana, established in 2004. This was followed by the Centre for Animal Welfare and Ethics at the University of Queensland in 2005. 

In addition to the single institution centres, international centres have been established, mostly to facilitate knowledge transfer of animal welfare science, such as the World Organisation for Animal Health (OIE) centres, or to devise legislation, such as the European Union (EU) centres. The OIE has established three centres of expertise in animal welfare for the international dissemination of animal welfare knowledge in Europe (centre based in Teramo, Italy, established in 2004), Asia, the Far East, Oceania (centre based in New Zealand and Australia, established in New Zealand in 2007; the Australian centres joined in 2008) and the Americas (established in Uruguay and Chile in 2009; Mexico joined in 2013). The European Union recently established Reference Centres [8]—initially for pigs, poultry and other small farmed animals—in 2019 and 2020. These are consortia of academic institutions aimed at assisting in the enforcement of EU legislation on animal welfare, through training courses and knowledge transfer. The relevance and impact of these aggregations, which include animal welfare in their titles, remain unclear, but with their increasing numbers, it is timely to examine their role. 

We conducted a bibliometric search to examine the output of publications on animal welfare from animal welfare centres and compare it with that not from such centres, as well as to improve our understanding of the relevance of explicit animal welfare citations in the names of research and teaching groups. Our hypothesis was that centres including animal welfare in their names are more likely to be animal centred in their scientific publications about animal welfare.

## 2. Method 

Searches were made using Web of Science, one of the world’s most popular databases that is publisher-independent and provides access to many different databases. This database excludes early access articles that have not been assigned a volume number or page number. The main search was for articles published between 1900 and the present (7 May 2020), searching by address with the search string “allati jolet or altaya alhaywaniye or animal welfare or animal wellbeing or bem estar animal or bem-estar animal or buenastarea animalelor or benessere animale or benessere degli animale or bienestar animal or bien-être animal or dierenwelzijn or djuerskydd or dobobit zivotinja or dobrostanu or dobrych zivotnich or duong fuli or dyrevelferd or dzivnieku labturibas or elainten hyvinvointa or gyvunu gerove or hayvan nafaki or loomade heaohu or pidminek zvirat or tierschutz or wohlbefinden tier”. This included the following languages: Hungarian, Russian, English, Portuguese, Romanian, Italian, Spanish, French, Dutch, Swedish, Croatian, Polish, Chinese, Norwegian, Danish, Latvian, Finish, Lithuanian, Turkish, Estonian, Czech and German. Even though best efforts were employed to make the list of terms as inclusive as possible, it may not be exhaustive. In addition, journal indexing in Web of Science is not evenly distributed across different countries, which means that our search was not able to include publications from an array of scientific journals in languages other than English. The Web of Science data resulting from this search were analysed using the Web of Science software for the type, year of publication, origin in terms of organisation and country, language, funding agency, author, subject, and publication source. 

A sample of animal welfare publications was used to estimate the proportion produced by authors affiliated with an animal welfare institutional centre, denoted originally as a centre or other similar term, and the proportion produced by authors not affiliated to such a group. Thus, a second search included the above terms in the subject or title of the articles but included or excluded them from the address. It was recognised that not all publications by centres or similar groupings would have these terms in the abstracts or titles of the articles. The proportion of these articles produced in centres was calculated. The proportion of funding sources related to the animal industries was compared between the two searches using a Chi square test. 

## 3. Results

### 3.1. The First Literature Search of Publications Including the “Animal Welfare” Term in Their Addresses

In the initial search, a total of 4851 entries were detected that included the “animal welfare” term in their addresses. 

**Figure 1:**
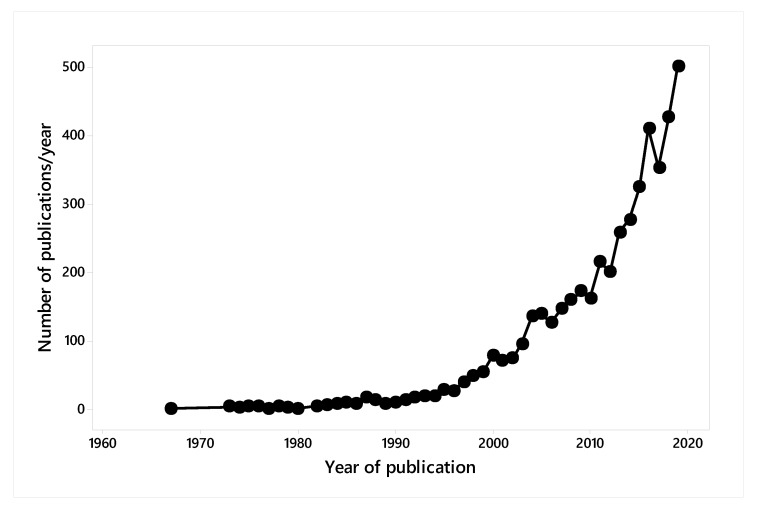
Publications from centres including the term “animal welfare” or its translation into major languages between 1995 and 2019.

These entries were classified into categories that were not mutually exclusive. Of the 4851 entries, 3947 were articles, 264 proceedings papers, 245 reviews, 206 editorial material, 173 book chapters, 167 letters, 126 meeting abstracts, 15 early access, 14 book reviews, 13 corrections, 10 notes, two biographical items, two discussions, and one was poetry. The years of publication are shown in Figure 1, with the first publications in 1964 but with publications remaining at a very low level until 1995, then increasing gradually until 2005, then remaining approximately consistent until 2012, followed by a further increase to 2019 (Figure 1). 

**Figure 2:**
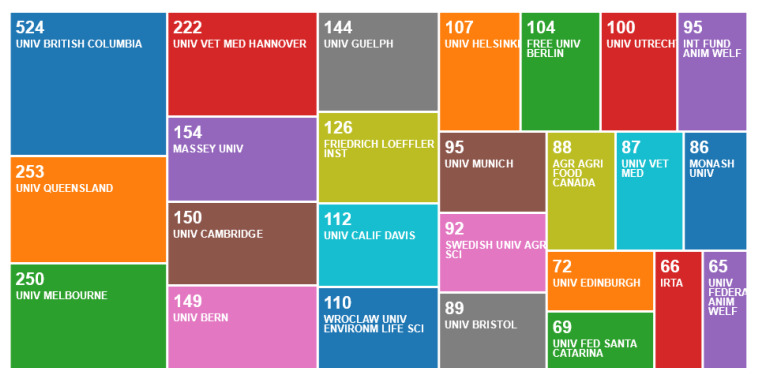
Number of publications per organisation, according to an online survey of publications by animal welfare centres in the Web of Science, May 2020.

The organisations with over 200 publications from a single centre in the first search came from just three countries, Canada, Australia and Germany; other organisations with smaller outputs came mostly from Europe (Figure 2).

Some countries achieved high numbers of centre publications from having multiple centres, most notably England, the USA and Italy, whereas Germany, Canada and Australia achieved high numbers with only one or two centres. 

**Figure 3:**
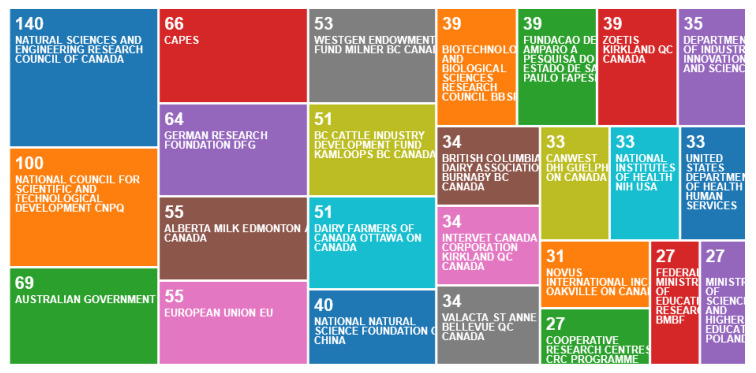
Number of publications per funding source, according to an online survey of publications by animal welfare centres in the Web of Science, May 2020.

The funding sources (Figure 3) supporting this work came principally from Canada, a diverse range of industry and government sources (10 of 25 sources funded most publications, including the Natural Sciences and Engineering Council, Alberta Milk Edmonton AB, Westgen Endowment Fund, BC Cattle Industry Development Fund, and Dairy Farmers of Canada). Other notable sources included Australia’s Government and Cooperative Research Centres (CRCs, co-operatively funded by government and animal industries), German Research Foundation, Brazil’s CNPq (Brazilian National Research Council) and CAPES (Coordination of Superior Level Staff Improvement), the National Natural Science Foundation of China and the UK’s BBSRC. Eleven of the 25 top funding agencies were industry based, with the Australian funding source, CRC, counted as industry based. 

**Figure 4:**
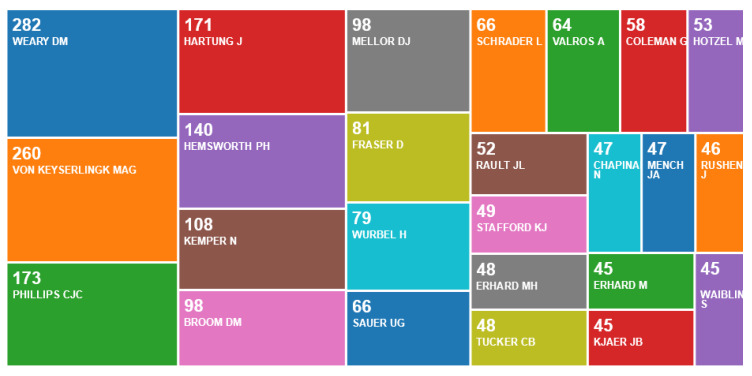
Number of publications per author, according to an online survey of publications by animal welfare centres in the Web of Science, May 2020.

The principal authors with more than 100 publications were from Canada (Weary, D.M., n = 282; Von Keyserlingk, n = 260), Australia (Phillips, C.J.C., n = 173; Hemsworth, P.H., n = 140) and Germany (Hartung, J., n = 171) (Figure 4).

**Figure 5:**
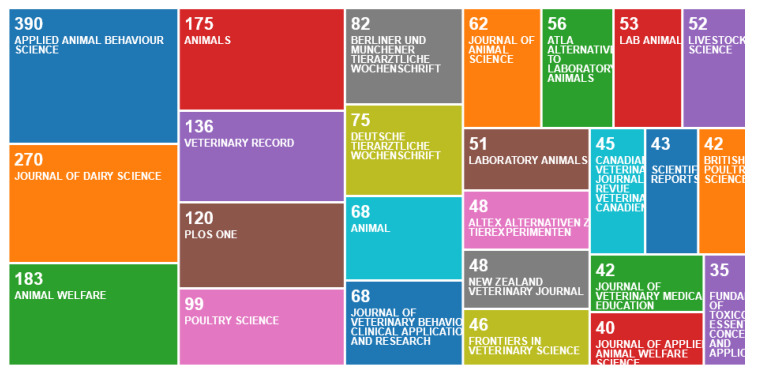
Number of publications per publication channel, according to an online survey of publications by animal welfare centres in the Web of Science, May 2020.

Relatively few publication channels were used (Figure 5), with only six scientific journals publishing more than 100 articles (Applied Animal Behaviour Science, Journal of Dairy Science, Animal Welfare, Animals, Veterinary Record and PLoS One). 

**Figure 6:**
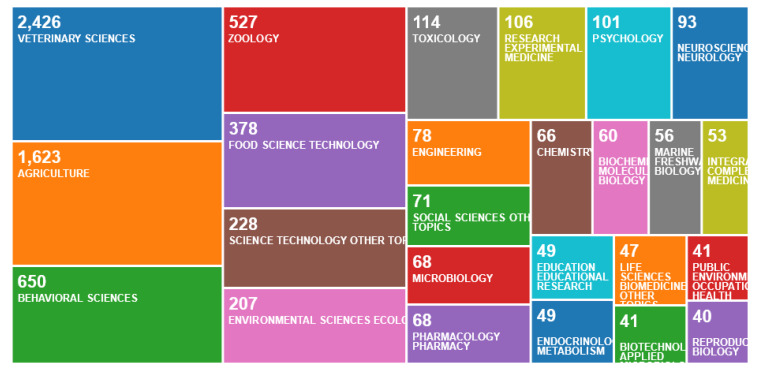
Number of publications per subject classification, according to an online survey of publications by animal welfare centres in the Web of Science, May 2020.

Nearly all (4404/4851 or 91%) were in English, with 373 in German, 46 in Polish, 11 in Spanish, 10 in Portuguese, 3 in Hungarian, and 2 each in Dutch and French. The subject of the work (Figure 6) was most commonly classified as the veterinary sciences, then agriculture, the behavioural sciences, zoology, food science technology, science technology (other topics), the environmental sciences/ecology, toxicology and research experimental medicine. 

The mean citations per item was 14, with a total of 67,957 citations (57,414 without self-citations) in 38,730 articles (36,078 without self-citations).

### 3.2. The Second Literature Search, of Animal Welfare Publications that Did or Did Not Include the “Animal Welfare” Term in Their Addresses

**Figure 7:**
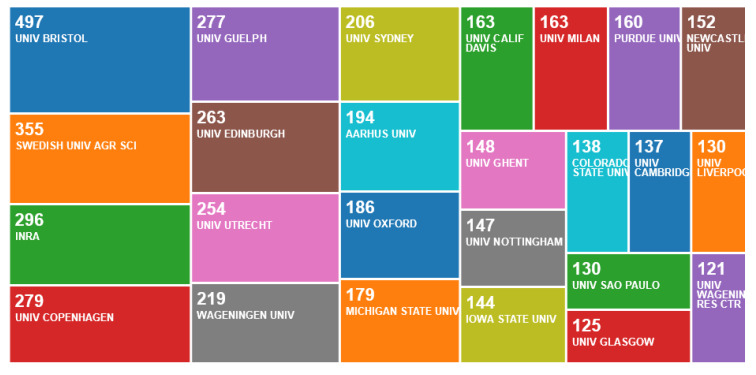
Number of publications per organisation, according to an online survey of animal welfare publications not by animal welfare centres in the Web of Science, May 2020.

In the second search, a total of 21,139 entries were detected that included the “animal welfare” term in their titles or abstracts. When the “animal welfare” term was excluded from the address, this number declined to 19,497. Thus, for this second search, the exclusion of the “animal welfare” term from the address caused the exclusion of 1642 entries, which means that in this sample, approximately 7.8% of the publications were produced in centres of animal welfare. A significant output (> 200 publications) of publications without the “animal welfare” term in their addresses was evident from the University of Bristol, Swedish University of Agricultural Science, INRA, University of Copenhagen, University of Guelph, University of Edinburgh, University of Utrecht, Wageningen University and University of Sydney (Figure 7). 

**Figure 8:**
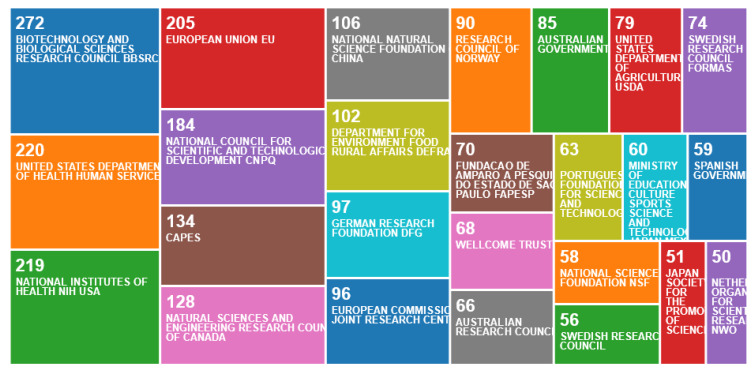
Number of publications per funding agency, according to an online survey of animal welfare publications not by animal welfare centres in the Web of Science, May 2020.

A different set of funding sources was evident in publications without the “animal welfare” term in their addresses, only one of which included industry funding (if Australian Research Council funding is considered industry based), in contrast to the funders of centres, which frequently included industry sources. The difference in funding sources, 1 out of 25 in this search and 11 out of 25 in the first search, was statistically significant (Chi square = 10.96, *p* < 0.001). The most notable were the UK government’s BBSRC, the US Department of Health Services, the National Institutes of Health, the USA, the National Council for Scientific and Technological Development, the EU, the Brazilian government’s CAPES, the National Sciences and Engineering Research Council of Canada, the National Natural Science Foundation, China and the UK government’s Department of Environment, Food and Rural Affairs, DEFRA (Figure 8). All other funding sources with > 50 publications were government sources, except the Wellcome Trust. 

**Figure 9:**
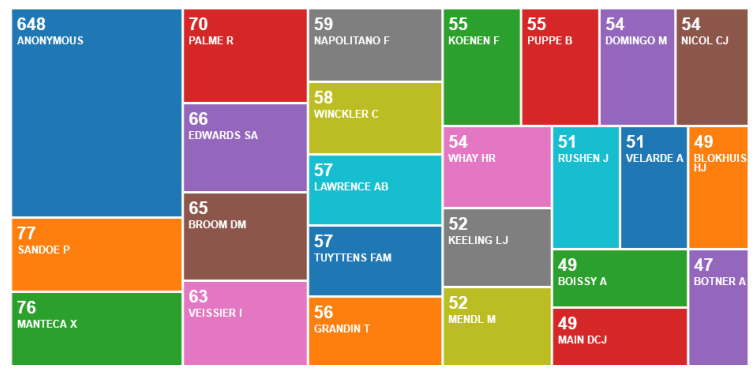
Number of publications per author, according to an online survey of animal welfare publications not by animal welfare centres in the Web of Science, May 2020.

New authors emerged that differed from those in centres (Figure 9). Numbers per author were lower than for the publications from centres. The key authors with more than 50 publications were Sandoe, P.; Manteca, X.; Palme, R.; Edwards, S.A.; Broom, D.M.; Vessier I.; Napolitano F.; Winckler, C.; Lawrence, A.B.; Tuyttens, F.A.M.; Grandin, T.; Koenen, F; Whay, H.R.; Keeling, L.J.; Mendl, M.; Rushen, J.; and Velarde A. This search included many new items and much editorial material that was anonymous. 

## 4. Discussion

The understanding of the relevance and impact of the organisation of animal welfare research activities into centres faces an initial limitation, related to the variety of formats and names for these centres. For instance, the University of Bristol maintains an “animal welfare and behaviour” research group, but this is not recognised in the group’s publications, which include instead the Centre for Behavioural Biology, an interdisciplinary venture between three Schools. This important group of animal welfare researchers appeared in our search under the classification “publications on animal welfare not including a centre in the address”. Thus, the interpretation of our results requires caution; nonetheless, our review did show some interesting characteristics that may contribute to the understanding of the impact of animal welfare centres, as well as indicate possible priorities to further the development of this research field. The fact that a small percentage of animal welfare scientific publications are identifiable as coming from centres that include animal welfare in their names suggests an important opportunity to increase the identity of this research field. This may relate to the scarcity of studies in identity across research groups, although identity research is well developed in many aspects [9]. It may be not so relevant for traditional research fields, such as physiology, nutrition or genetics, but identity building for more recent scientific fields, such as animal welfare, seems relevant for robust and faster development, proper recognition and the fulfilment of the initial goal: the improvement of animals’ lives.

The results for researchers with more than 100 papers suggest that countries with few centres supported some highly productive scientists, presumably because of the greater demand for work focused on a few scientists. The observed varying contribution of centres to research output suggests that there are different characteristics of welfare centres. Some might be primarily related to research, others clinic-focused, and others teaching or short course focused, as is typically required from university faculties—to work on teaching, outreach and research, a long-recognized challenge [10]. The variation may also be related to the different conditions for research output in different institutions, as well as the different levels of funding. In other words, grouping into a centre does not shield research from the regular obstacles faced by scientists in general. 

A source of the variation in the support for centres between countries is the political will to create and develop a centre. Some countries have more concern for animal welfare issues, as demonstrated by the World Animal Protection’s Animal Protection Index [11]. Some, particularly those in Europe, have whole parties dedicated to justice for animals. In these countries, there is more likely to be the political desire to support the creation and activities of an animal welfare centre. If there is a focus on animal welfare by advocacy groups, a government may see an advantage in developing a centre that can support research-based solutions to animal welfare issues. 

There seems to be important advantage for the development of a research direction, creating centres with a clear identification of the field of research. This is suggested by the fact that there are many institutions with animal welfare centres but with widely varying output. An advantage is that a common approach within a centre may be more easily developed, and the centre may become recognised as a brand by outside personnel and organisations. This, in turn, may lead to the recognition of a corporate approach, with benefits to students and staff working in challenging areas, as well as recognition and mutual support for those working within this common approach in other institutions. In addition, the development of a group identity may foster expectation and the recognition of expertise in the field, as identity provides definitional, descriptive, justificatory and prescriptive information, which constructs cognitive, emotional, behavioural and social boundaries, rendering the sense of self more comprehensible and practicable [12]. This self-identity may be further acknowledged by governments, philanthropic organisations and individuals, the scientific community, advocacy groups, and industry groups, which, in turn, is likely to encourage support and funding from these groups. Both the increases in funding and recognition of expertise tend to draw other scientists into the area.

Another positive contribution of establishing a centre may be related to more permanent lines of research in animal welfare within an institution, since individual initiatives are likely to depend on single researchers. In this case, if the researcher leaves the institution, no open path for continuing this field of studies is obvious. However, if a centre is established, if the researcher leaves one institution and establishes a new centre when starting in a new institution, a single researcher may impact the structure of animal welfare science research in more than one institution, thus leaving an open institutional path for newcomers, who do not have to start from scratch. 

The type of approach may be considered a relevant issue in animal welfare research, in the sense that it may be animal centred or non-animal centred. As per the demand of animal welfare science, considering Harrison’s landmark book [2] and the animal protection movement, it seems logical that animal welfare science should be animal centred. The initial hypothesis of this work, that centres including “animal welfare” in their names were more likely to be animal centred, was challenged by the results observed, since there was more industry-sourced funding for centres, which in turn may be associated with human- or at least mixed-centred approaches. This issue is a priority for the advancement of animal welfare that is coherent with the original motivation for this scientific field and, as such, warrants further studies with specific goals related to the potential of animal welfare publications to enhance the lives of animals, in a combined or stand-alone format, that necessarily stems from a sincere effort to represent the interests of the animals. Unfortunately, it is frequent to observe that the expected benefits to the animals themselves may not be achieved, due to a relative appropriation of animal welfare terminology by initiatives that fail to put the focus on the animals. Some examples are animal welfare certification programs, which fail to improve the lives of farm animals [13], and similar failures within multinational and international corporate social responsibility standards [14]. Further research on how important the animal point of view is within the scientific literature on animal welfare seems warranted. 

The difficulties for a centre to maintain the focus on decreasing animal suffering and improving opportunities for positive feelings may be related to the evident need for the inclusion of animal ethics aspects, as an essential basis for good animal welfare science. In any field of work, the importance of philosophy for good science is recognised in at least four points: the clarification of scientific concepts, the critical assessment of scientific assumptions or methods, the formulation of new concepts and theories, and the fostering of dialogue between different sciences as well as between science and society [15]. From these, it is easy to realise how important animal ethics is for animal welfare science, which developed in the first place as a response to societal demands [16]. Even though the concepts of animal welfare and animal ethics are not the same, it is reasonable to think of them as inseparable [17,18]. As for the importance of plainly evident centre names, we do find animal welfare research centres that include the term ethics in their names, such as the Animal Welfare Science and Bioethics Centre (Massey University) and the Centre for Animal Welfare and Ethics (University of Queensland). This representation seems positive and may indicate a future trend in the area. 

An increase in animal ethics content is desirable. General knowledge on animal welfare scientific literature suggests that most centres focus on welfare issues, without the involvement of ethical decision making. If animal welfare science is, as proposed by Fraser [16], a mandated science stemming from societal concerns regarding animals, it is inextricably based on animal ethics. Animal ethics is the field for discussions on how to protect animals from suffering, as specific animal welfare knowledge so accurately does, but also from other problems, such as disrespect for their bodily integrity or disregard for their dignity. For example, the anthropocentric concept of unnecessary suffering [19] should be addressed, since the opinion on which suffering is necessary for farm animals to endure has historically been profit centred. Another issue is how to support the publication and enactment of laws such as the Animal Protection Act in Switzerland, which obliges the consideration of the protection of animal welfare and dignity [20]. Questions must be considered regarding what life expectancies we owe to animals, as when it fits human purposes, we kill them soon after birth, for example, in the cases of male chicks in the egg industry or male calves in the dairy industry. Thus, animal welfare centres may better address societal needs when they discuss, in an animal-centred fashion, the need for changes in the acceptability status of animals, i.e., what is it acceptable to do to an animal? What are humans or countries doing and, perhaps more urgent to address, what are they not doing to protect animals [21]? 

Some pitfalls in relation to the increased recognition of a centre may require attention. With time, members of an established centre may be perceived as elitist, as the leader in the field in such a manner that may be associated with a certain degree of isolation from the rest of the academic community, which is not good for science. Even though this may also occur with individual researchers, we expect a higher risk for an organised group. In addition, it may be more difficult for scientists working outside of the centre to interact with its members, so it is beneficial for centres to make constant efforts regarding external collaborations. Regarding the generation of ideas, there may be a similar risk, with the possible exclusion of views that do not concur with those adopted by the centre.

The key features of successful centres appear to be (1) independence from industry, advocacy and government biases; (2) a willingness to collaborate externally and internally; (3) a good support network within the university; (4) value placed on independence from industry, advocacy groups, government; and (5) a strong reliance on science based approaches. The main contribution of animal welfare centres within universities and other research institutions seems to rely on the latter; however, this is best if accompanied by ethical reflections, which is clear from the current understanding of the inseparability of science and ethics in animal welfare [17]. To work on the important and omnipresent issues regarding animal ethics, the centres benefit from inputs and preferably permanent collaborations with thinkers within the humanities. 

### Do the Advantages Outweigh the Disadvantages?

Notwithstanding the recognition of the importance of animal welfare science and education [22,23] and the clear recommendation for animal welfare teaching [24], animal welfare is not yet a mainstream discipline in universities. However, it is one of the most common sources of concern amongst the public and an area of knowledge for which future professionals are very likely to be demanded by society and, as a consequence, a variety of employers. Centres may encourage those in the university to contribute to a common cause and become more involved in welfare research than they otherwise would have been. Thus, an additional advantage of the existence of an animal welfare centre is probably the fact that it may naturally induce animal welfare teaching, in a manner that may be more specific than when groups do not have explicitly mention animal welfare in their names.

The reliance on industry sources for funding was a key feature of some of the leading centres, i.e., those in countries where there were only one or two centres. This incurs a risk that industry will seek to influence the outcomes of the research, either directly by only allowing the publication of results that they approve of or indirectly by maintaining a close relationship with key researchers, whose grant funding is dependent on them adopting the approach that industry favours [25,26]. However, the relationship may go both ways; the researchers may also be able to influence industry and its approach to animal welfare. A close relationship between research centres and industry is also positive since it tends to facilitate the process of constantly adopting knowledge as it is produced. In the case of animal welfare, this may be extremely positive for the animals involved in each specific industrial activity. This close relationship therefore has advantages and disadvantages. Requirements for industry to make funding available only through open competition with the independent review of applications may be considered by governments, particularly if—as is the case with the Australian and UK governments, at least—the industry funding is matched by government funding, which is public money. Double blind procedures may also be required. This may help animal welfare to progress more rapidly towards standards approved by the public and decrease the risks of industry trying to influence animal welfare researchers to defend production systems that are not supported by the public. 

Some guidance for the management and focus of centres seems worthwhile. These include the independence of the publications of the centre from outside influences, a focus on animal welfare issues, the use of open access to make the material available to the public wherever possible, the avoidance of harm to the animals used in research and the centralised management of publicity for the centre’s output. Special attention is required for centres to maintain the main focus on the animals, since there are a variety of pressures for an increased emphasis on human-centred approaches. While we recognise the importance of a holistic approach, considering non-human animals, humans and environmental issues in a One Welfare conceptualization [27,28], the attribution of animal welfare science to understand the ethically-relevant subjective experiences of animals is important [17], which, in turn, can only be achieved through an animal-centred approach. Thus, for the use of the term “animal welfare” in the name of the group to facilitate the consolidation of animal welfare as a scientific discipline, bringing it to the fore in a more direct manner, some level of standardization and the consideration of basic animal centred criteria seem necessary. 

Finally, international centres, such as those of the OIE and EU, may present relevant contributions if they are properly resourced. For instance, an important initiative presented by the OIE is the proposal of a Global Animal Welfare Strategy [29], with a worldwide recommendation for four pillars in an animal welfare strategy: the development of animal welfare standards; capacity building and education; communication with governments, organisations and the public; and the implementation of animal welfare standards and policies. The international animal welfare centres, logically, depend on adequate support to be able to achieve such broad and complex objectives. A permanent challenge for animal welfare centres is the ubiquitous presence of competing interests. Thus, it is highly relevant that all centres be supported to work emphasizing scientific approaches, being as free as possible from the various political and economic pressures common to issues related to animal welfare in general, as well as those related specifically to farm animal welfare.

## 5. Conclusions

This work contributes to the knowledge in animal welfare science by providing an updated overview of research and teaching centres that are active in animal welfare science across the world, with some description of their scientific output as well as other relevant particularities. The importance and advantages of naming such centres with the inclusion of the term “animal welfare” were evident. On the other hand, as our initial hypothesis was challenged by the results, some level of standardization and a consideration of basic animal centred criteria for the use of the term “animal welfare” in centres is welcome, with the goal of ensuring the representation of the animals’ point of view within animal welfare science, since this was the very reason for its development. In some countries, most notably Canada and Australia, it appears that only a small number of centres have emerged, with usually one or two that are largely funded by industry. This incurs the risk of a lack of independence. If publications come from scientists that are not in centres or from multicentre countries, the funding sources are more likely to be government-based.
